# Investigation of *BTLA* tagging variants with risk of esophagogastric junction adenocarcinoma

**DOI:** 10.1042/BSR20191770

**Published:** 2019-12-13

**Authors:** Weifeng Tang, Shuchen Chen, Mingqiang Kang, Jun Liu, Chao Liu

**Affiliations:** 1 Department of Cardiothoracic Surgery, Affiliated People’s Hospital of Jiangsu University, Zhenjiang, Jiangsu Province, China; 2 Department of Thoracic Surgery, Fujian Medical University Union Hospital, Fuzhou, Fujian Province, China; 3 Department of Medical Oncology, Fujian Medical University Cancer Hospital and Fujian Cancer Hospital, Fuzhou, Fujian Province, China

**Keywords:** BTLA, adenocarcinoma, Immune, Lymphocyte, Polymorphism

## Abstract

**Background:** Variants in *B- and T-lymphocyte attenuator* (*BTLA*) gene are likely to affect the function of BTLA protein.

**Methods:** In the present case–control study, we selected *BTLA* tagging single-nucleotide polymorphisms (SNPs) (rs16859629 T>C, rs1982809 G>A, rs2171513 G>A and rs3112270 A>G) and conducted a case–control study to identify the association of *BTLA* SNPs with risk of esophagogastric junction adenocarcinoma (EGJA). The present study involved 1236 new incident EGJA cases and 1540 cancer-free controls.

**Results:** The genotypes of *BTLA* SNPs were analyzed using a SNPscan Kit. No association was also found between the *BTLA* SNPs and the susceptibility of EGJA in overall comparsion. In subgroup analyses, the *BTLA* rs1982809 was found to be associated with an increased susceptibility of EGJA (AA versus GG: OR^adjusted^ = 2.09, 95% CI 1.08–4.07, *P* = 0.030; and AA versus GA/GG: OR^adjusted^ = 1.99, 95% CI 1.04–3.82, *P* = 0.039). In haplotype comparison, we identified that TAAG haplotype with the order of *BTLA* rs16859629, rs1982809, rs2171513 and rs3112270 SNPs might increase the susceptibility of EGJA (OR = 3.07, 95% CI = 1.41–6.71; *P* = 0.003).

**Conclusion:** To conclude, the present study suggests that *BTLA* T_rs16859629_A_rs1982809_A_rs2171513_G_rs3112270_ haplotype may increase the susceptibility of EGJA. More studies should be conducted to evaluate whether *BTLA* polymorphisms may influence the susceptibility of cancer in the future.

## Introduction

The morbidity of esophagogastric junction adenocarcinoma (EGJA) is promoting rapidly, both in developing and developed contries [1–3]. EGJA comprises a vital portion esophageal and gastric cancer, with an increasing ratio. It is reported that EGJA is a common fatal tumor in China. EGJA is regarded as an entity with a specific clinical feature and molecular profile. The potential protective factor or a real cause of EGJA is unclear. Thus, an understanding of the potential risk factors influencing the development EGJA biology may be helpful to diagnosis and prognostic assessment for the supervision of EGJA patients.

During the activation of T lymphocytes, they can express some receptors for receiving various signals. B- and T-lymphocyte attenuator (BTLA), also named CD272, is a most recently identified and studied member of the immune globulin (Ig) superfamily [4–7]. BTLA is a glycoprotein and it contains two tyrosine-based inhibitory motifs [8]. During activation, BTLA is not expressed on T helper type 2 (Th2) cells, but Th1 cells. The expression of BTLA on T cells participates in negative regulation of T cell and then leads to an decreased T-lymphocytes proliferation [9]. Recently, many investigations have focused on the relationship of BTLA with inflammation, autoimmune disease and cancer. Shi et al. reported that BTLA-herpes virus entry mediator (HVEM) checkpoint axis might be implicated in the regulation of inflammation in liver [10]. A previous study indicated that the up-regulation of BTLA gene expression and soluble BTLA (sBTLA) was validated in thymoma-associated myasthenia gravis [11]. A prognostic investigation showed that the levels of immune checkpoints sBTLA could be considered as a biomarker for unresectable pancreatic adenocarcinoma cases with a poor survival [12]. A functional study identified that IFN-γ level in circulating T-lymphocytes could be promoted by inhibiting BTLA/HVEM pathway [13]. Additionally, Feng et al. [14] and Lan et al. [15] reported that the level of BTLA expression in gastric carcinoma (GC) might be a useful biomarker for the evalution of GC prognosis.

Single-nucleotide polymorphisms (SNPs) in *BTLA* gene are likely to affect the role of BTLA protein. Some studies have kept a watchful eye on the correlation of *BTLA* variants with the development of cancer [16–18]. Fu et al*.* reported that the frequencies of *BTLA* rs1844089 and rs2705535 SNPs may alter the risk of breast cancer [17]. In Polish population, it was found that *BTLA* rs1982809 G>A, a 3′-UTR SNP, might be a low-penetrating risk factor for the development of renal cell carcinoma [18]. In addition, another study indicated that *BTLA* rs1982809 G and rs2705511 C alleles were more frequent in patients with chronic lymphocytic leukemia compared with healthy controls [16]. In view of the vital role in cancer development and progress, we supposed that *BTLA* SNPs might be correlated with EGJA susceptibility. Here, *BTLA* tagging SNPs (rs16859629 T>C, rs1982809 G>A, rs2171513 G>A and rs3112270 A>G) were selected. The aim of the present study was to identify the association of *BTLA* tagging SNPs with risk of EGJA.

## Materials and methods

### Subjects

The present study involved 1236 new incident EGJA patients and 1540 cancer-free controls. Among these patients, 393 cases patients diagnosed with EGJA and treated at two affiliated hospitals of Fujian Medical University [Union Hospital (Fuzhou, China) and Fujian Cancer Hospital (Fuzhou, China)] from January 2014 to June 2018. In addition, 843 patients with EGJA were from Jiangsu University People’s Hospital (Zhenjiang, China) from January 2008 to June 2018. Siewert type was used in our study [19]. Here, all EGJA cases included were Siewert type II (their centre within 1 cm proximal and 2-cm distal of the anatomical cardia). All included EGJA cases were diagnosed at the first time with histopathological test. For EGJA cases, the major included criteria were: (a) individuals who did not have a history of other cancers, (b) without any immunological diseases and (c) EGJA patients were not treated wtih any chemotherapy and/or radiotherapy before the enrolment. We recruited 1540 cancer-free subjects as controls matching to the EGJA patients by sex, year of birth (±5 year) and ethnicity (Eastern Chinese Han nationality). They were from the hospitals mentioned above for regular health examination. The major included criteria for controls were: (a) cancer-free individuals, (b) without any immunological diseases, (c) sex and age matching to EGJA cases and (d) Han nationality who living in Eastern China. Each patitcipant signed a consent form. The experimental protocol was authorized by the ethics committees of the Jiangsu University.

### Selection of SNPs

The tagging SNPs of *BTLA* [from 112458030 to 112504757 in chromosome 3 (extending 5 Kb, upstream and downstream, respectively)] were structured and collected from Chinese populations via Genome Variation Server data. The criteria of tagging SNPs selection were described in our previous studies [20,21].

### DNA extraction

Genomic DNA was extracted from the colletced blood samples with the Promega DNA Kit (Promega, Madison, U.S.A.), according to the explanatory memorandum. A 2-μl DNA was droped in NanoDrop ND-1000 spectrophotometer (Wilmington, U.S.A.) to evaluate concentration and purity of DNA sample.

### Genotyping

The genotypes of *BTLA* rs16859629, rs1982809, rs2171513 and rs3112270 SNPs were analyzed using a SNPscan Kit (Genesky Biotechnologies Inc., Shanghai, China) as described previously [22–24]. PCR process was conducted in a 20-μl mixture volume in 96-well plates. ABI 3730xl DNA Analyzer was used to identify the genotype. The data of the sequencing were read by GeneMapper 4.1 (AppliedBiosystems, U.S.A.). One hundred and eleven DNA specimens were randomly chosen for repeat genotyping by another person in a blind fashion, and the obtained variants were concordant.

### Statistical method

For each locus in *BTLA* gene, an online *χ*^2^ test was used to assess the Hardy–Weinberg equilibrium (HWE) [25]. The Student *t* test was performed to deal with continuous variables of demographic characteristics between two groups. And *χ*^2^ test was harnessed to handle the categorical variables (e.g., age, sex, cigarette using and alcohol consumption) and variant distributions of *BTLA* SNPs between two groups. The haplotypes of *BTLA* gene were evaluated by SHESIS software [26]. Odds ratios (ORs) and 95% confidence intervals (CIs) were used to evaluate the strength of the correlation of *BTLA* SNPs with the risk of EGJA. Multiple logistic regression analysis was harnessed to check the distribution of *BTLA* rs16859629, rs1982809, rs2171513 and rs3112270 genotypes between two groups. Subgroup analyses between the *BTLA* variants and characteristic variables were also conducted. The adjusted *P* values, ORs and 95% CIs were calculated by adjustment for age, sex, cigarette using and drinking. A *P* < 0.05 (two-way tests) was defined as significance in all statistical tests. All statistical analyses described previously were performed in SAS 9.4 software (SAS Institute Inc., Cary, NC, U.S.A.). Using PS software (http://biostat.mc.vanderbilt.edu/twiki/bin/view/Main/PowerSampleSize), the power value (*α* = 0.05) was calculated [27,28]. We also used the false-positive report probability (FPRP) to determine the significant findings [29].

## Results

### Baseline characteristics

Table 1 summarizes age, sex, cigarette using and alcohol consumption in two groups. EGJA patients had a mean age of 64.28 ± 8.64 years. The age and sex ratio was not significant between two groups (*P* = 0.408 and *P* = 0.485, respectively). The success rate of *BTLA* rs16859629, rs1982809, rs2171513 and rs3112270 genotyping was of high quality (>97%) (Table 2). We pressented the data of minor allele frequency (MAF) in Table 2. In control group, the frequencies of genotype distribution met HWE (Table 2).

**Table 1:** Distribution of selected demographic variables and risk factors in this case–control study

Variable	Overall cases (*n* = 1236)		Overall controls (*n* = 1540)		*P*^1^
	*n*	%	*n*	%	
Age (years)	64.28 (±8.64)		64.17 (±10.32)		0.775
Age (years)					0.408
< 64	568	45.95	732	47.53	
≥64	668	54.05	808	52.47	
Sex					0.485
Male	885	71.60	1084	70.39	
Female	351	28.40	456	29.61	
Smoking status					0.087
Never	884	71.52	1146	72.73	
Ever	352	28.48	394	27.27	
Alcohol use					**<0.001**
Never	1,028	83.17	1359	88.25	
Ever	208	16.83	181	11.75	

1 Two-sided *χ*^2^ test and Student’s *t* test.

**Table 2:** Primary information for *BTLA* targging SNPs (rs2171513 G>A, rs3112270 A>G, rs1982809 G>A and rs16859629 T>C)

Genotyped polymorphisms	rs2171513 G>A	rs3112270 A>G	rs1982809 G>A	rs16859629 T>C
Chr	3	3	3	3
Position_38	112466080	112461797	112463893	112471533
Region	3′-UTR	Promoter	3′-UTR	intron_variant
MAF^1^ in database (1000 g Chinese Han populatons)	0.188	0.269	0.216	0.067
MAF in our controls (*n* = 1540)	0.196	0.280	0.256	0.084
*P* value for HWE^2^ test in our controls	0.625	0.114	0.796	0.898
% Genotyping value	98.34%	98.56%	98.52%	97.48%

1 MAF, minor allele frequency.

2 HWE, Hardy–Weinberg equilibrium.

### Relationship of *BTLA* rs16859629, rs1982809, rs2171513 and rs3112270 SNPs with EGJA

The genotype distributions and frequencies of *BTLA* rs16859629, rs1982809, rs2171513 and rs3112270 genotypes are presented in Table 3. In a single SNP analysis, the *BTLA* rs2171513 G>A genotype frequencies were 62.83% (GG), 32.67% (GA) and 4.50% (AA) in EGJA patients and 63.70% (GG), 32.27% (GA) and 4.03% (AA) in the cancer-free controls. When the *BTLA* rs2171513 GG genotype was defined as the reference, the *BTLA* rs2171513 GA genotype was not correlated with the susceptibility for EGJA (GA versus GG: adjusted OR = 1.04, 95% CI: 0.88–1.22, *P* = 0.668); the *BTLA* rs2171513 AA genotype was not correlated with the susceptibility for EGJA (AA versus GG: adjusted OR = 1.23, 95% CI: 0.83–1.81, *P* = 0.302). In addition, the *BTLA* rs2171513 GA/AA genotypes did not conferred the risk to EGJA in the dominant model (GA/AA versus GG: adjusted OR = 1.06, 95% CI: 0.90–1.24, *P* = 0.497). In the recessive genetic compared model, when the *BTLA* rs2171513 GG/GA genotypes were defined as a reference, the *BTLA* rs2171513 AA genotype was not correlated with susceptibility for EGJA (AA versus GG/GA: adjusted OR = 1.21, 95% CI: 0.83–1.78, *P* = 0.327) (Table 3). No association was also found between *BTLA* rs3112270 A>G, rs1982809 G>A and rs16859629 T>C SNPs and the susceptibility of EGJA (Table 3).

**Table 3:** Logistic regression analyses of associations between *BTLA* targging SNPs (rs2171513 G>A, rs3112270 A>G, rs1982809 G>A and rs16859629 T>C) and the risk of EGJA

Genotype	EGJA case (*n* = 1236)		Controls (*n* = 1540)		Crude OR (95%CI)	*P*	Adjusted OR^1^ (95% CI)	*P*
	*n*	%	*n*	%				
rs2171513 G>A								
GG	754	62.83	985	64.38	1.00		1.00	
GA	392	32.67	489	31.96	1.05(0.89–1.23)	0.580	1.04(0.88–1.22)	0.668
AA	54	4.50	56	3.66	1.26(0.86–1.85)	0.241	1.23(0.83–1.81)	0.302
GA+AA	446	37.17	545	35.62	1.07(0.91–1.25)	0.404	1.06(0.90–1.24)	0.497
GG+GA	1146	95.50	1,474	96.34	1.00		1.00	
AA	54	4.50	56	3.66	1.24(0.85–1.82)	0.269	1.21(0.83–1.78)	0.327
A allele	500	20.83	601	19.64				
rs3112270 A>G								
AA	639	52.99	782	51.11	1.00		1.00	
AG	472	39.14	641	41.90	0.90(0.77–1.06)	0.197	0.90(0.77–1.06)	0.192
GG	95	7.88	107	6.99	1.09(0.81–1.46)	0.582	1.10 (0.82–1.48)	0.538
AG+GG	567	47.02	748	48.89	0.93(0.80–1.08)	0.330	0.93(0.80–1.08)	0.333
AA+AG	1111	92.13	1423	93.01	1.00		1.00	
GG	95	7.88	107	6.99	1.14(0.85–1.52)	0.380	1.15(0.86–1.53)	0.343
G allele	662	27.45	855	27.94				
rs1982809 G>A								
GG	668	55.44	846	55.29	1.00		1.00	
GA	461	38.26	586	38.30	1.00 (0.85–1.17)	0.964	1.00(0.85–1.17)	0.984
AA	76	6.30	98	6.41	0.98(0.72–1.35)	0.911	1.00(0.85–1.37)	0.980
GA+AA	537	44.56	684	44.71	0.99(0.85–1.16)	0.941	1.00(0.86–1.16)	0.979
GG+GA	1129	93.70	1432	93.59	1.00		1.00	
AA	76	6.30	98	6.41	0.98(0.72–1.34)	0.917	1.00(0.73–1.36)	0.983
A allele	613	25.44	782	25.56				
rs16859629 T>C								
TT	1028	85.74	1265	83.94	1.00		1.00	
TC	158	13.18	231	15.33	0.84(0.68–1.05)	0.122	0.84(0.67–1.04)	0.106
CC	13	1.08	11	0.73	1.45(0.65–3.26)	0.363	1.39(0.62–3.13)	0.426
CT+CC	171	14.26	242	16.06	0.87(0.70–1.08)	0.197	0.86(0.70–1.07)	0.166
TT+CT	1186	98.92	1496	99.27	1.00		1.00	
CC	13	1.08	11	0.73	1.49(0.67–3.34)	0.332	1.43(0.64–3.21)	0.389
C allele	184	7.67	253	8.39				

1 Adjusted for age, sex, smoking, status of Chronic hepatitis B virus infection and drinking.

Bold values are statistically significant (*P* < 0.05).

### Relationship of *BTLA* rs16859629, rs1982809, rs2171513 and rs3112270 SNPs with EGJA in subgroup analysis

Table 4 presents the variant frequencies of *BTLA* rs1982809 SNP in stratification analysis. When we conducted an adjustment for gender, age and alcohol consumption, we identified that the *BTLA* rs1982809 G>A was associated with an increased susceptibility of EGJA for ever smokers (AA versus GG: adjusted OR = 2.09, 95% CI 1.08–4.07, *P* = 0.030; and AA versus GA/GG: adjusted OR = 1.99, 95% CI 1.04–3.82, *P* = 0.039). We found that there was no significant association between *BTLA* rs1982809 G>A SNP and the risk of EGJA in other subgroups.

**Table 4:** Stratified analyses between *BTLA* rs1982809 G>A polymorphism and EGJA risk by sex, age, smoking status and alcohol consumption

Variable	*BTLA* rs1982809 (Case/Control)^1^			Adjusted OR^2^ (95% CI); *P*				
	GG	GA	AA	GG	GA versus GG	AA versus GG	GA/AA versus GG	AA versus (GG/GA)
Sex								
Male	488/605	328/412	49/61	1.00	0.99(0.82–1.20); *P*: 0.925	1.01(0.68–1.49); *P*: 0.981	0.99(0.83–1.19); *P*: 0.937	1.01(0.68–1.49); *P*: 0.966
Female	180/241	133/174	27/37	1.00	1.02(0.75–1.37); *P*: 0.916	0.99(0.58–1.69); *P*: 0.983	1.01(0.76–1.34); *P*: 0.932	0.99(0.59–1.66); *P*: 0.962
Age								
<64	304/391	205/287	40/51	1.00	0.92(0.73–1.17); *P*: 0.502	1.00(0.64–1.56); *P*: 0.996	0.93(0.75–1.17); *P*: 0.553	1.04(0.67–1.60); *P*: 0.876
≥64	364/455	256/299	36/47	1.00	1.07(0.86–1.33); *P*: 0.545	0.97(0.61–1.53); *P*: 0.896	1.06 (0.86–1.30); *P*: 0.609	0.94(0.60–1.48); *P*: 0.801
Smoking status								
Never	487/606	325/424	50/81	1.00	0.95(0.79–1.15); *P*: 0.600	0.78(0.54–1.14); *P*: 0.199	0.93(0.77–1.11); *P*: 0.392	0.80(0.56–1.15); *P*: 0.229
Ever	181/240	136/162	26/17	1.00	1.13(0.83–1.54); *P*: 0.449	**2.09(1.08–4.07);** ***P*: 0.030**	1.22(0.91–1.64); *P*: 0.193	**1.99(1.04–3.82);** ***P*: 0.039**
Alcohol consumption								
Never	563/737	378/524	63/90	1.00	0.95(0.80–1.13); *P*: 0.543	0.92(0.66–1.30); *P*: 0.639	0.94(0.80–1.11); *P*: 0.493	0.94(0.68–1.32); *P*: 0.725
Ever	105/109	83/62	13/8	1.00	1.40(0.91–2.17); *P*: 0.126	1.56(0.61–3.99); *P*: 0.350	1.42(0.94–2.16); *P*: 0.098	1.37(0.54-3.44); *P*: 0.504

1 The genotyping was successful in 1205 (97.49%) EGJA cases, and 1530 (99.35%) controls for *BTLA* rs1982809.

2 Adjusted for age, sex, smoking status and alcohol consumption (besides stratified factors accordingly) in a logistic regression model.

No association was found between the *BTLA* rs2171513 G>A, rs3112270 A>G and rs16859629 T>C SNPs and the susceptibility of EGJA in subgroup analyses (data was not shown).

### SNP haplotypes

Using haplotype constructing software mentioned above [26], we observed 12 *BTLA* gene haplotypes. We identified that TAAG haplotype with the order of *BTLA* rs16859629, rs1982809, rs2171513 and rs3112270 SNPs might increase the susceptibility of EGJA (OR = 3.07, 95% CI = 1.41–6.71; *P* = 0.003). However, other observed *BTLA* gene haplotypes did not alter the susceptibility of EGJA (Table 5).

**Table 5:** BTLA haplotypes frequency (%) and the association between BTLA haplotypes and risk of EGJA

Haplotypes	Case		Control		Crude OR (95%CI)	*P*
	*n*	%	*n*	%		
TGGA	1159	48.64	1459	48.41	Reference	
TAGG	407	17.08	518	17.19	0.99(0.85–1.15)	0.887
TGAA	283	11.88	350	11.61	1.02(0.85–1.21)	0.843
CGGA	136	5.71	175	5.81	0.98(0.77–1.24)	0.856
TGAG	120	5.04	154	5.11	0.98(0.76–1.26)	0.88
TGGG	71	2.98	105	3.48	0.85(0.62–1.15)	0.309
TAGA	70	2.94	87	2.89	1.01(0.73–1.40)	0.938
TAAA	69	2.9	79	2.62	1.10(0.79–1.53)	0.575
CAGG	34	1.43	57	1.89	0.75(0.49–1.16)	0.192
TAAG	22	0.92	9	0.3	**3.07(1.41–6.71)**	**0.003**
CAGA	7	0.29	19	0.63	0.46(0.19–1.11)	0.076
Others	5	0.21	2	0.07	3.15(0.61–16.26)	0.149

With the order of *BTLA* rs16859629 T>C, rs1982809 G>A rs2171513 G>A and rs3112270 A>G in gene position.

### Power calculation and FPRP determining

Using PS software (http://biostat.mc.vanderbilt.edu/twiki/bin/view/Main/PowerSampleSize), the power value (α = 0.05) was calculated [27,28]. For *BTLA* rs1982809 G>A SNP, the power value was 0.631 in AA versus GG genetic model and 0.589 in AA versus GG/GA genetic model among ever smokers. In haplotype comparison, T_rs16859629_A_rs1982809_A_rs2171513_G_rs3112270_ haplotype could increase the susceptibility of EGJA (power value, 0.830).

## Discussion

The incidence of EGJA is increasing in both the East and Western countries. It is reported that altered lifestyle and lower chronic Helicobacter pylori infection may result in an increasing incidence of EGJA [30,31]. The etiology of EGJA may be attribute to gene and environment factors. Recent evidence suggested that the variants of immune and inflammatory response related genes could alter the risk of cancer [21,32–35]. Considering an important role of *BTLA* gene in immune, we chose *BTLA* tagging SNPs (rs16859629, rs1982809, rs2171513 and rs3112270) and explored their effects on the development of EGJA. Here, we identified that *BTLA* TAAG haplotype with the order of rs16859629, rs1982809, rs2171513 and rs3112270 SNPs might be associated with the development of EGJA.

*BTLA* rs1982809 G>A SNP locates in 3′-UTR, which could participate in post-transcriptional control. Recently, studies have been conducted to identify a potential effect of *BTLA* rs1982809 locus on the development of malignancy. *BTLA* rs1982809 polymorphism, a 3′-UTR SNP, was found to be associated with the development of renal cell carcinoma in Polish populations [18]. Another case–control study also found that *BTLA* rs1982809 polymorphism were associated with chronic lymphocytic leukemia [16]. Subsequently, in the same study, the funcional investigation demonstrated that the presence of *BTLA* rs1982809 G allele was correlated with lower expression *of BTLA* mRNA in lymphocyte as compared with rs1982809 A allele [16]. In the present study, we first studied the relationship between *BTLA* rs1982809 locus and cancer risk in Asians. We found this SNP might not alter the overall EGJA risk. However, *BTLA* rs1982809 locus was identified as a risk factor to EGJA in smoking subgroup, which was similar to the previous reports [16,18]. The results suggested that the role of *BTLA* rs1982809 G>A polymorphism may be influenced by environmental factors. However, the subjects included in smoking subgroup were related small, these findings may be underpowered. In the future, more case–control studies should be conducted to evaluate whether *BTLA* rs1982809 G>A polymorphism might inhibit the function of B and T cells and influence the susceptibility of cancer.

In the present case–control study, the *BTLA* haplotypes were also constructed. We found *BTLA* T_rs16859629_A_rs1982809_A_rs2171513_G_rs3112270_ haplotype might influence the risk of EGJA. However, this rare *BTLA* haplotypes only altered the susceptibility of a minor fraction of the EGJA patients. We first expolre the association of *BTLA* haplotypes with cancer risk in Asians. Our findings should be verified in the future studies.

It is necessary to acknowledge the limitations in the present case–control study. First, the present study was designed as hospital-based. Although the frequencies of genotype distribution in *BTLA* rs16859629, rs1982809, rs2171513 and rs3112270 SNPs met HWE and the MAFs of these selected SNPs in control group were close to the database for Chinese, the bias might have happened. Second, we only included four risk factors (gender, age, smoking and alcohol consumption). And other potential environment factors (e.g. body mass index, intake of vegetable and fruit, education level and economic income) were not considered. Thus, the potential interactions between gene and these environment factors could not addressed. Third, the participants included were related small in some subgroups, the observations may be insufficient evidence to identify a relationship with a definitive power. Fourth, in the present study, the biological functions of *BTLA* SNPs were not studied. Finally, only four *BTLA* tagging SNPs (rs16859629, rs1982809, rs2171513 and rs3112270) were selected, which could not fully assess the total hereditary susceptibility in *BTLA* gene.

To conclude, this investigation suggests that *BTLA* T_rs16859629_A_rs1982809_A_rs2171513_G_rs3112270_ haplotype may increase the susceptibility of EGJA. More studies with multiple environment factors should be carried out to evaluate whether *BTLA* variants may influence the susceptibility of cancer in the future.

## Abbreviations

FPRP: false-positive report probability
MAF: minor allele frequency
SNP: single-nucleotide polymorphism
